# Human Immunodeficiency Virus-Type 1 LTR DNA contains an intrinsic gene producing antisense RNA and protein products

**DOI:** 10.1186/1742-4690-3-80

**Published:** 2006-11-08

**Authors:** Linda B Ludwig, Julian L Ambrus, Kristie A Krawczyk, Sanjay Sharma, Stephen Brooks, Chiu-Bin Hsiao, Stanley A Schwartz

**Affiliations:** 1 Division of Allergy, Immunology and Rheumatology, Department of Medicine, School of Biomedical Science and Medicine, State University of New York at Buffalo, Buffalo, New York 14203, USA; 2 Department of Surgery, School of Biomedical Science and Medicine, State University of New York at Buffalo, Buffalo, New York 14203, USA; 3 Division of Infectious Disease, Department of Medicine, School of Biomedical Science and Medicine, State University of New York at Buffalo, Buffalo, New York 14203, USA; 4 Present address: 2519 145th Circle, Vancouver, Washington 98686, USA

## Abstract

**Background:**

While viruses have long been shown to capitalize on their limited genomic size by utilizing both strands of DNA or complementary DNA/RNA intermediates to code for viral proteins, it has been assumed that human retroviruses have all their major proteins translated only from the plus or sense strand of RNA, despite their requirement for a dsDNA proviral intermediate. Several studies, however, have suggested the presence of antisense transcription for both HIV-1 and HTLV-1. More recently an antisense transcript responsible for the HTLV-1 bZIP factor (HBZ) protein has been described. In this study we investigated the possibility of an antisense gene contained within the human immunodeficiency virus type 1 (HIV-1) long terminal repeat (LTR).

**Results:**

Inspection of published sequences revealed a potential transcription initiator element (INR) situated downstream of, and in reverse orientation to, the usual HIV-1 promoter and transcription start site. This antisense initiator (HIVaINR) suggested the possibility of an antisense gene responsible for RNA and protein production. We show that antisense transcripts are generated, *in vitro *and *in vivo*, originating from the TAR DNA of the HIV-1 LTR. To test the possibility that protein(s) could be translated from this novel HIV-1 antisense RNA, recombinant HIV antisense gene-FLAG vectors were designed. Recombinant protein(s) were produced and isolated utilizing carboxy-terminal FLAG epitope (DYKDDDDK) sequences. In addition, affinity-purified antisera to an internal peptide derived from the HIV antisense protein (HAP) sequences identified HAPs from HIV+ human peripheral blood lymphocytes.

**Conclusion:**

HIV-1 contains an antisense gene in the U3-R regions of the LTR responsible for both an antisense RNA transcript and proteins. This antisense transcript has tremendous potential for intrinsic RNA regulation because of its overlap with the beginning of all HIV-1 sense RNA transcripts by 25 nucleotides. The novel HAPs are encoded in a region of the LTR that has already been shown to be deleted in some HIV-infected long-term survivors and represent new potential targets for vaccine development.

## Background

Certain viruses employ bidirectional transcription. The human dsDNA virus, Herpes simplex, produces latency-associated transcripts that are antisense to a portion of the regulatory protein, ICP0, mRNA [1]. The human herpes virus, Varicella zoster, contains bidirectional promoters for transcription of the DNA polymerase and major DNA-binding protein mRNAs. These promoters are coordinately activated by a shared site for a cellular transcription factor, USF[2]. Plant viruses, such as the ssDNA tomato leaf curl virus, can encode multiple, overlapping open-reading frames (ORFs) and utilize either strand of the genomic ssDNA or a complementary DNA intermediate as templates for transcription [3]. There is evidence for bidirectional promoters in the hepadnaviruses, woodchuck hepatitis virus and the hepatitis B virus[4,5]. The human hepatitis D virus, which possesses a single, circular genomic RNA, translates two of its major proteins (hepatitis delta antigen(s)) from a complementary anti-genomic RNA intermediate[6]. The strategy of encoding genes on either strand of dsDNA, or genomic RNA or ssDNA and complementary nucleic acid intermediate, enables viruses to greatly enhance coding capability.

The prevailing model of HIV-1 transcription, however, has been of a single viral promoter located in the 5' long terminal repeat (LTR) of the dsDNA proviral intermediate, which allows transcription to occur only with the production of positive-strand polarity transcripts, i.e., sense transcripts. These transcripts are variably spliced and translated or remain unspliced to allow production of the HIV-1 proteins and genomic RNA, respectively[7]. Only a few papers have suggested bidirectional transcription for HIV-1[8,9]. Michael, *et al*. found evidence for negative-strand RNA transcripts in mononuclear cells from 15 HIV-1 infected patients. They identified a negative-strand promoter in the U3 region (-78 to -172 from the HIV-1 cap site), and isolated a cDNA from HIV-1 infected cells capable of encoding an antisense ORF in a region overlapping the retroviral envelope gene [8]. However, no protein from this region was isolated.

With the discovery of an antisense gene in the human T-cell leukemia virus type 1 encoding the HBZ factor that interacts with other transcription factors, the presumption that antisense genes in the human retroviruses cannot exist or matter should be dispelled [10-13]

**Figure 1:**
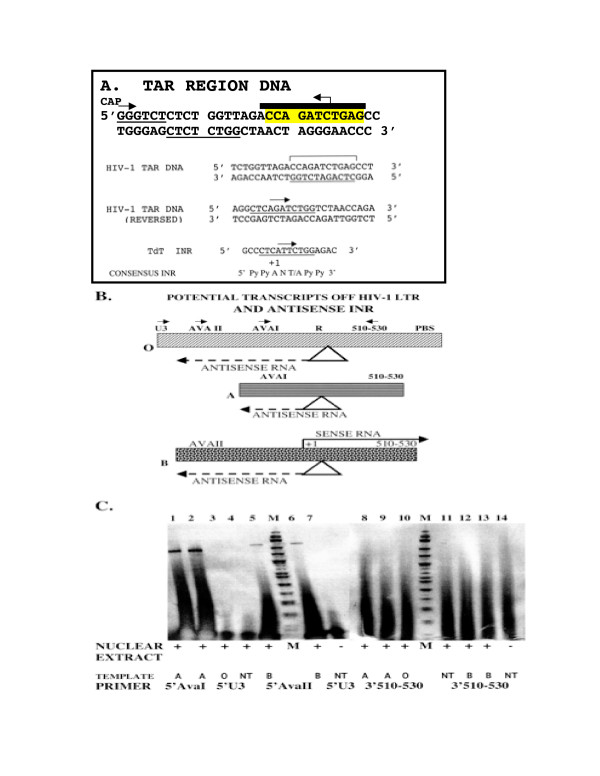
Identification of an antisense transcript originating from the HIV-1 antisense INR (HIVaINR). (**A**) The TAR sequence in HIV-1 DNA from the cap site (start of sense transcription) to +59 is shown. Sequence comprising the HIVaINR is indicated with a heavy overbar and reverse arrow, and this region of sequence (reversed) is compared to the terminal deoxynucleotidyl transferase (TdT) INR element [14] and the consensus INR[15]. Underlined sites in the HIV-1 TAR DNA indicate location of putative sense INR elements [19]. (**B**) Gel-purified DNA templates used for the generation of transcripts from the HIV-1 promoter and the HIVaINR. The original template is labeled "O" (650 bp) and contains the entire LTR from U3 through the PBS. Two additional truncated templates are designated "A"(270 bp) and "B"(350 bp). (Not to scale.) The primers U3, AvaII, and AvaI (forward arrows) and 510–530 (reverse arrow) are indicated above the original template (O). (**C**) Primer extension of purified RNA generated *in vitro *using the above HIV-1 LTR templates and *Drosophila *embryo nuclear extracts. cDNA products were produced from an antisense RNA annealing to a biotinylated sense 5'AvaI primer (213 bp, lanes 1 and 2) or biotinylated sense 5'AvaII primers (274 bp, lanes 5 and 6), but not with a biotinylated sense 5'U3 primer (lane 3), as demonstrated by denaturing PAGE, Southern transfer and colorimetric detection (method adapted from U.S. patents 5,900,358; 5,919,677; 6,392,029). The same RNA samples were analyzed for the presence of HIV-1 sense transcripts using a biotinylated antisense primer (3'510–530) (lanes 8–14 correspond to lanes 1–7, respectively), but yielded no transcripts of the expected size (97 bp). This experiment was performed three times with the same results. M lanes received biotinylated ¡-X174 markers with bands from 24–460 bp shown. NT = no template control.

The possibility of partially overlapping, and bidirectional HIV-1 promoters is suggested by our discovery of an antisense initiator (HIVaINR) in the TAR region of the viral LTR (Figure 1A). Initiator elements (INR) were originally described as alternative core promoter elements to the TATA box, the highly conserved sequence for RNA polymerase II transcription initiation [14]. Remarkably, the HIVaINR is located 18–28 nucleotides downstream from the usual start site of HIV-1 transcription, and is oriented to promote an antisense transcript that is opposite in direction and complementary to the beginning ~25 nucleotides of all HIV-1 mRNAs (Figure 1A). The HIVaINR complementary sequence matches the proposed consensus initiator sequence derived by analyzing a series of mammalian gene initiators and comparing over 500 promoter sequences[15,16]. Because of this homology, we postulated that if the HIVaINR functioned, it could indicate the presence of a novel HIV antisense gene responsible for either an RNA or protein product.

We demonstrate that this HIVaINR functions as an initiation site for antisense RNA transcription in *in vitro *transcription reactions and *in vivo*, in human cells that have stably incorporated the HIV-1 LTR sequences. Using a recombinant HIV-1 antisense gene-FLAG vector transfected into human cells, we also show that protein products can be translated. Implicit in these results is that translational recoding event(s) must be occurring because of the presence of intervening stop codons preceding the carboxyterminal sequence encoding the FLAG epitope (DYKDDDDK) used for antibody detection and immunoaffinity purification. Finally, we show that affinity-purified antibody to an internal peptide derived from the HIV antisense protein (HAPs) sequence specifically recognizes the expected molecular weight proteins in human HIV+ peripheral blood lymphocyte lysates.

## Results

### A consensus sequence suggested a HIV-1 antisense initiator element

Many eukaryotic promoters contain either a TATA box or an initiator (INR), or both, as a core promoter element that serves to direct transcription initiation[17]. The TATA box functions to direct transcription initiation approximately 30 base pairs (bp) downstream, whereas an INR is a sequence motif that encompasses and orients the transcription start site, generally from a conserved internal C A (+1) [14,16,17]. Importantly, an INR can act independently to direct the assembly of transcription complexes that recruit RNA polymerase II. The terminal deoxynucleotidyl transferase (TdT) INR determines the position of the start site of transcription in the absence of other core promoter elements (ibid, above). Extensive mutagenesis and analysis of the function of variant sequence INRs suggested an approximate consensus INR of 5' Py Py A (+1) N T/A Py Py 3' (Figure 1A) (ibid, above). We observed the presence of a sequence in HIV-1 TAR region DNA, 5'CCAGATCTGAG3', that when reversed, 5'CT**CA**GATCTGG3', was remarkably similar to the strong TdT INR consisting of the sequence 5' CT**CA**^+1^TTCTGG3' (Figure 1A)[14]. This reversed HIV-1 sequence also contained the preferred internal **CA**^+1 ^N T/A and was surrounded by several pyridimines (C,T), shown by mutational and experimental analysis to function as INR elements and matching the loose, but consistent consensus INR (Figure 1A) (ibid, above). Because an INR also imparts directionality to the recruited transcriptional complex, as well as directing the start site for transcription, the HIV-1 antisense INR (HIVaINR) sequence, 5' AGATCTGAG3' could be predicted to indicate an initiation site for transcription in the opposite direction, or antisense that of usual HIV-1 transcription. Inspection of this HIVaINR sequence in TAR revealed that it was completely conserved in the 20 B strain HIV-1 complete genomes listed[18]. We were therefore interested to examine whether this site functioned as a region of transcription initiation in the context of adjoining promoter sequences in the LTR. Inasmuch as genes can be responsible for either RNA or RNA/protein products, this could be the signal for a novel *antisense gene within the HIV LTR*.

### Detection of the antisense transcript in *in vitro *transcription reactions

The HIV-1 LTR contains three putative initiator (INR) elements within the first 50 bp of the R region or TAR DNA (Figure 1A). Two INRs previously described are oriented in the sense direction and are located at the cap site and downstream of the transcription start site (Figure 1A, underlined)[19]. The HIV-1 TATA box is upstream of these two INRs and would be expected to enhance the functioning of even a weak INR element. The HIVaINR is located inbetween these two sites, but is oriented to start transcription in the opposite direction (Figure 1A, overbar, reverse arrow). We examined whether antisense transcripts were specifically produced off the HIV-1 LTR using an *in vitro *transcription reaction system (Figure 1, and described in U.S. patent 5,919,677). A variety of truncated fragments of the HIV-1 LTR were used as templates in these *in vitro *transcription reactions (diagrammed in Figure 1B, and not shown). *Drosophila *nuclear extracts contain RNA polymerases and transcription factors previously shown to interact effectively with INR elements and were used in the transcription reactions with the LTR templates[20,21]. Primer extension or RNase digestion was used to map the origins of the purified RNAs synthesized *in vitro *from the designated HIV-1 LTR templates (Figure 1B, 1C). The expected size cDNA following primer extension for an antisense transcript originating from the HIVaINR was clearly identified using two separate biotin-labeled primers, as shown in Figure 1C (lanes 1,2, or 5,6 with the expected size products of 213 and 274 bp, respectively). As a control, the same in vitro synthesized and purified RNA samples were simultaneously assayed for sense RNA transcripts by primer extension and no cDNA product was observed (Figure 1C: lanes 8–10,12,13: expected size product would be 97 bp). Thus the putative sense orientation INR elements were not functioning in this system. However, they can be viewed as mutant internal control INRs within the same LTR DNA with the sequence surrounding the HIV-1 transcription start site, 5'CTGGGTCT 3', and at a site 37 nucleotides from the start site with the sequences 5'CTCTCTGG 3' (Figure 1A, brackets)[19]. Except for a 3 bp internal deletion, the latter sequence is identical to the reversed HIVaINR (Figure 1A). Additional controls included reactions with no template (NT) and no nuclear extract (lanes 7 and 14) or simply no template (lane 4), in which only the biotinylated primer was detected (Figure 1C). This indicated that the nuclear extract was not contributing any of the product bands observed.

These results suggested a preferential accumulation of the *Drosophila *transcription factors (and RNA polymerase) at the HIVaINR over the two putative sense INRs and identified for the first time a novel antisense promoter element within HIV-1 TAR region DNA. In addition, these experiments identified a single HIV antisense transcript originating from the region of the HIVaINR. If there had been multiple initiation sites off the R-U3 regions of the LTR, one would have expected to see additional product bands in these experiments. No additional bands suggesting alternative start sites were observed with any of the HIV-1 LTR templates studied. RNase digestion, performed simultaneously with primer extension, confirmed these results (not shown). These experiments also revealed that the 3' end of the antisense transcript in the same samples was not detected by the complementary U3 primer (Figure 1C, lane 3).

### Detection of the HIV-1 antisense transcript *in vivo*, in human cell lines that were either transiently transfected or stably transfected with the HIV-1 LTR

**Figure 2:**
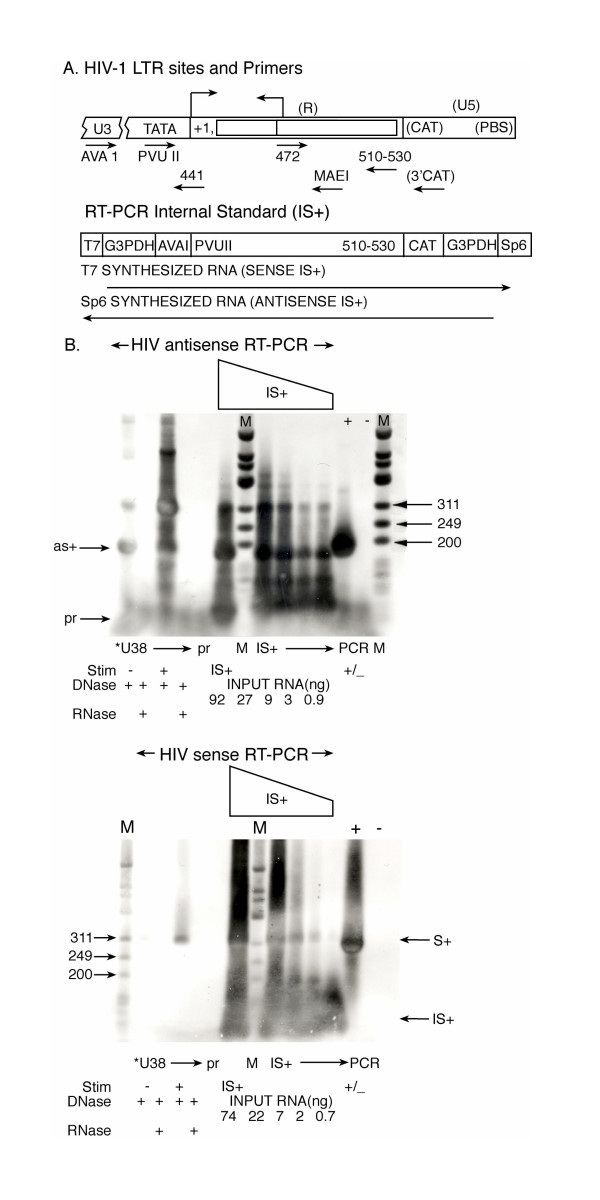
Comparison of antisense RNA transcripts generated *in vivo *from the HIVaINR with HIV-1 sense transcripts in the absence of Tat protein. (**A**) Map of landmarks in the HIV-1 LTR and the relative location of primers used for RT-PCR (not to scale). The DNA template used to synthesize the HIV-1 sense and antisense RNA internal standard IS+ is also diagrammed. (**B**) Analysis of *in vivo *transcripts from U38 cells, which have stably incorporated HIV-1 LTR-CAT sequences, clearly demonstrates intrinsic antisense RNA transcripts. Total RNA was obtained from U38 cells grown with and without transient stimulation with PMA and Ca ionophore (Stim +/-). U38 RNA samples were split and subjected to DNase treatments or DNase and RNase treatments, as indicated. Each sample was then analyzed by non-radioactively labeled RT-PCR for the presence of HIV-1 antisense RNA (expected product 183 bp) and for the presence of HIV-1 sense transcripts (expected size product 285 bp). Each set of RT-PCR reactions also included a titration of the internal standard IS+ RNA subjected to the identical RT-PCR reaction, as well as a primer alone control (pr), in which no RNA template was added. The IS+ titration is indicated above the lanes. Additional band(s) in the HIV antisense RT-PCR with stimulation (+) could represent antisense RNA self-primed with a 5' terminal hairpin and extending to predicted pseudoknot motifs (expected size 312, ~500). M lanes received biotinylated ø-X174 HinfI markers with bands from 24–726 bp in size. Triplet open arrowheads indicate 311, 249, and 200 bp in M, with the single arrow indicating antisense RT-PCR product (labeled as+) and single arrowhead indicating sense RT-PCR product (labeled s+). PCR + and – lanes received pHIV-CAT or no template with the same primers used for the corresponding RT-PCR. This analysis was performed in triplicate with similar results.

The more telling question was whether HIV-1 antisense transcription could be detected *in vivo *with human cells susceptible to infection by HIV-1. In many previous systems it has been difficult to detect the presence of antisense transcripts in vivo despite apparent biological effects[22]. In preliminary experiments to determine the appropriate primers for use in RT-PCR, we used transient transfection of HIV-1 LTR-containing plasmids or controls into human Jurkat T cells (see additional File 2 (Figure 2S) from U.S. patent 6,392029). We had to redesign primers several times before finding regions that were specific only for the HIV LTR, and that did not also pick up intrinsic cellular sequences because of the shared transcription factor binding sites (not shown). For instance, primers overlapping the transcription factor binding site SP1 or the HIVaINR within the LTR also detected intrinsic cellular sequences, presumably because of the many human cellular genes also containing SP1 or INR sites (not shown). One of the primer sites that reliably detected only the antisense transcript was the complementary Ava I primer, used during the reverse transcription step (Figure 4). However, this site represents a region of considerable sequence heterogeneity in the HIV LTR and needed to be specifically designed with the HIV-1 strain in mind[18]. The second primer 441 was biotin-labelled and was added during the polymerase chain reaction (PCR). Biotin-labelled 441 was derived from sequence following the initial 5' terminal hairpin of the HIV-1 antisense transcript (Figure 4). This non-radioactive labelling modification of RT-PCR enhanced the sensitivity and specificity of detection (Figure 2B and Additional File 2). We employed rigorous internal controls for the RT-PCR, and compared the same total RNA samples following DNase treatment or DNase and RNase treatment, in order to ensure that we were studying RNA, and not a DNA contaminant.

**Figure 3:**
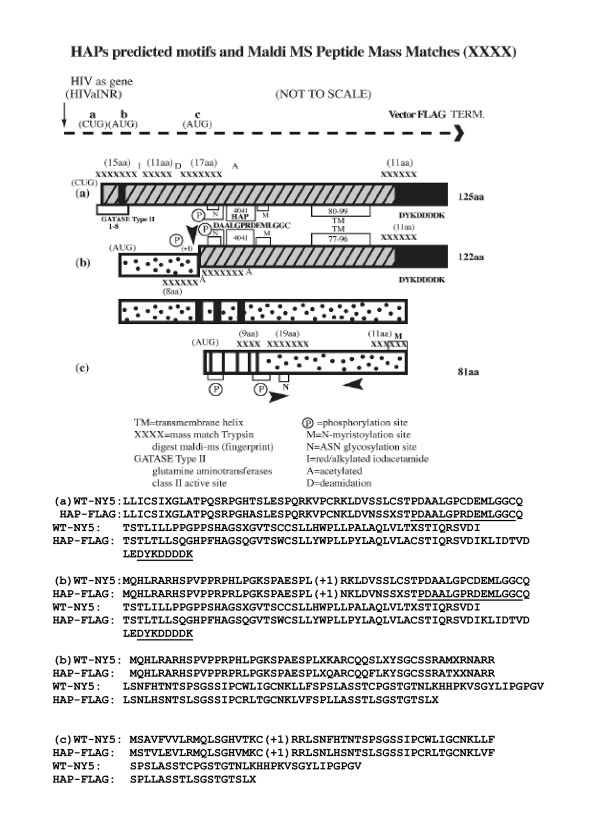
Diagram and predicted amino acid sequences of HIV-1 antisense proteins (HAPs). Discussed in text. Sites were detected using ScanProsite Tool [66]. HAP peptide 4041 and FLAG aa sequences are underlined.

**Figure 4:**
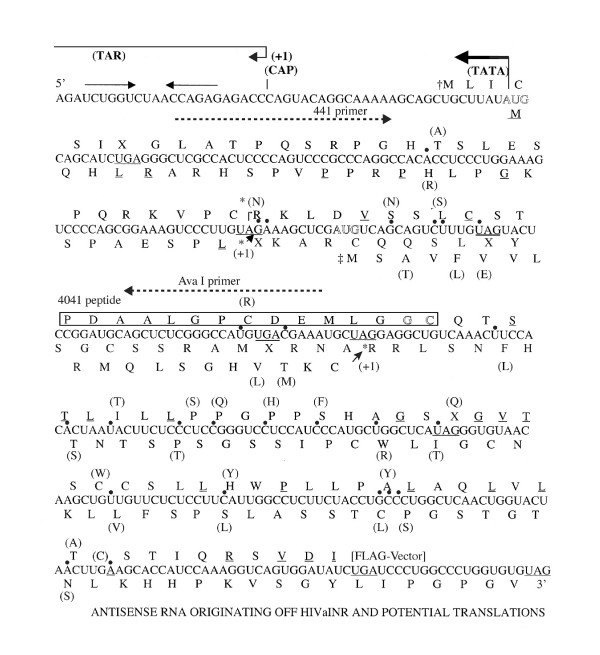
Diagram of antisense RNA derived from NY5 sequences originating from the HIVaINR, along with predicted amino acid sequence(s). The sequence varies from that of the HIV antisense gene inserted into HIV-AS-FLAG vector at the nucleotides indicated with a dot, and the alternative HAP amino acid is also indicated (discussed in text). Sites of postulated +1 ribosomal frameshifting are indicated with *. Stop codons are underlined and in the amino acid translation indicated with (X). The amino acids comprising the 4041 peptide are boxed, and the primers used for HIV antisense RNA detection are indicated (dashed arrows). Not shown is the run of four-five U bases embedded in a GC rich region encoded at the U3 terminus, which could potentially function as a termination signal (for either RNA polymerase 11 or 111) for this antisense RNA (compare B clade WEAU, or JRCSF with NY5) [18].

Many experiments indicated that the HIV-1 antisense RNA transcript is constitutively produced, with two examples given here (Figure 2B, and additional File 2). A single expected product band of 183 bp was observed following RT-PCR of purified RNA from Jurkat T cells transfected with pHIV-CAT vector containing the HIV-1 LTR (additional File 2, lanes 7a–12a)[23]. It was only observed with pHIV-CAT transfected cell RNA, but not mock transfected (no template, NT) or control Jurkat T cell RNA (additional File 2, lanes 1a–6a) indicating that intrinsic cellular RNA was not being detected. The expected product band was also only observed when the Jurkat T cells transfected with the HIV-1 LTR vector were amplified with the Ava 1 primer and the biotin-labeled 441 primer, but not with the Ava 1 primer and a biotin-labeled Mae 1 primer (additional File 2, compare lanes 7a–12a with 7b–12b). This indicated that the antisense RNA was initiating in the region of the HIVaINR inbetween the Mae 1 and 441 site as expected (diagrammed in Figure 2A). This also showed that the RNA samples were not contaminated with DNA from the transfected LTR vector (which would contain the Mae 1 site). Thus the HIV-1 antisense RNA originated between these two primer sites, situated 24 nucleotides apart in the HIV-1 TAR region DNA.

The HIV antisense transcript also initiated from the region of the HIVaINR in the stably-transfected U38 cells at levels comparable to or greater than HIV-1 sense transcripts in the absence of the HIV-1 Tat protein, as illustrated in Figure 2B. U38 is a human U937 derivative cell line containing stably integrated copies of the HIV-1 LTR promoter linked to the CAT gene[24]. The expected 183 bp size product band was observed with the HIV antisense RT-PCR (figure 2B, upper panel, arrow labeled as+). As well, HIV sense transcripts were produced in this cell line as indicated by the expected 283 bp product band in U38 RNA samples (HIV sense RT-PCR (figure 2B, lower panel). After stimulation with calcium ionophore and phorbol myristate ester (PMA), some increment in both sense and antisense transcription is observed (Figure 2B, *U38, Stim +). However, along with stimulation (and the production of more HIV-1 sense transcripts) additional antisense RT-PCR product bands are observed indicative of autopriming. One of these additional bands at 312 bp would be expected if the HIV-1 antisense RNA transcript extended from a 5' terminal hairpin to a predicted pseudoknot structure, for instance [25]. All primers were functioning effectively, as demonstrated by the RT-PCR Internal Standard RNA (IS+) controls, included in each analysis, as well as by PCR + controls (Figure 2B). The primers were not contaminated with template, as shown by the identical reaction mixtures receiving primer alone (labelled pr, Figure 2B). RNase treatment of the U38 RNA samples eliminated all the RT-PCR product bands, whether following HIV antisense RT-PCR or sense RT-PCR (Figure 2B, labelled RNase +). This confirmed the authenticity of the RNA synthesized in vivo, and analyzed by RT-PCR. The quality of the total RNA was also monitored by simultaneous analysis RT-PCR for intrinsic cellular G3PDH RNA transcripts, and additional controls performed included no reverse transcriptase added, and DNA contamination controls in each RT-PCR analysis (not shown). Similar results were obtained with BF24, a monocyte cell line that had been stably transfected with the HIV-1 LTR linked to the CAT gene, or with a cell line containing an HIV-1 truncated provirus, HL2/3, following transfection with pHIV-CAT [23,24].

### Translations of HIV-1 putative antisense ORF(s)

There were three possible start sites for translation from the intrinsic antisense RNA produced utilizing the HIV-1 LTR as template (Figure 3 and Figure 4). Each translational start was in a separate reading frame and contained at least one presumptive stop codon (diagrammed as a filled box, Figure 3). A short ORF initiating from an internal AUG could encode an 81 aa protein, if a +1 ribosomal frameshift occurred (Figure 3c and discussed in U.S. patent 6,392,029). The initial AUG encountered on the antisense RNA could initiate translation in another reading frame, and with a +1 ribosomal frameshift would generate a 105 aa protein (diagrammed in Figure 3b). Translation could also conceivably initiate at a CUG start codon as has been described for human fibroblast growth factor 2 [26]. Initiation of translation from non-AUG codons has also been described for HTLV-1[27]. This reading frame would generate a 108 aa protein in the wild-type virus and contained an early TGA stop codon (Figure 3a). The predicted amino acid sequences from each translation are shown in Figures 3 and 4. Translational recoding events such as read-through or ribosomal frameshifting through a stop codon are already utilized by retroviruses, including HIV-1 [7,28,29]. HIV-1 employs translational recoding in order to translate the gag-pol precursor polyprotein. In addition, given the appropriate downstream RNA structure, the TGA codon may actually code for selenocysteine [30]. The HIVaINR antisense RNA, in addition to a 5' terminal hairpin (Figure 4), has very extensive structure by Mfold analysis (not shown). Secondary/tertiary RNA structure of this intrinsically produced antisense RNA was already suggested by the RT-PCR experiments (Figure 2 and not shown). The presence of RNA structures such as hairpins or pseudoknots would suggest an inherent plasticity available for translational decoding [25,28,29].

### Proteins are translated from intrinsic HIV-1 antisense gene sequences inserted into a recombinant vector

**Figure 5:**
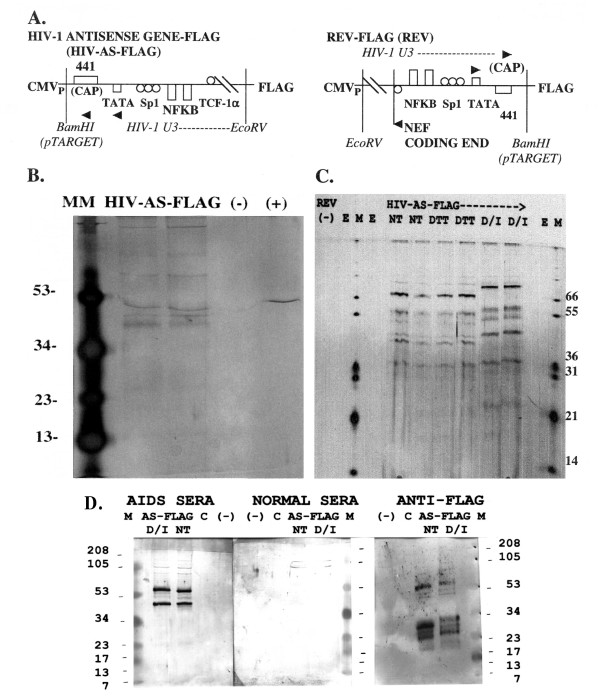
HIV-1 antisense gene proteins (HAPs) are translated within human cells. (**A**) Schematic diagram of recombinant vector containing the HIV-1 antisense gene sequences linked to carboxyterminal sequences encoding a FLAG epitope (**HIV-AS-FLAG**), or the same HIV-1 sequences in the opposite orientation linked to the same vector (**REV**). Landmarks in the HIV-1 LTR U3 region are not to scale. (**B**) HL2/3 cells were transfected with **HIV-AS-FLAG**, mock transfected **(-)**, or transfected with the identical vector containing a luciferase gene linked to FLAG **(+)**, and the cell lysates incubated on anti-FLAG immunoaffinity columns. Following incubation of the three lysates, each column was washed, and then "batch eluted". The eluates were then PEG concentrated and analyzed using Tris-tricine-urea SDS-PAGE with a mini-gel apparatus, followed by silver staining. MM is Multi-Mark multi-colored standard (Invitrogen). **(C) HIV-AS-FLAG **or **REV **(-) control transfected-cell lysates were incubated on anti-FLAG immunoaffinity columns, and the concentrated, pooled eluate fractions analyzed by Tris-tricine-urea SDS-PAGE (large gel apparatus) and silver staining. The HIV-AS-FLAG eluates were treated with DTT, with DTT and iodoacetamide (D/I), or received no treatment (NT), as indicated. E, empty; M, Mark 12 protein standard (Invitrogen). **(D) **Immunoblot analysis of HIV-AS-FLAG transfected cells. Cell lysates from HL2/3 cells transfected with HIV-AS-FLAG were affinity-purified over anti-FLAG M2 antibody resins, and the eluates were then concentrated (**AS-FLAG**). The eluates were split and subjected to either no treatment (NT) or treatment with DTT and Iodacetamide (D/I), and then analyzed using Tris-tricine-urea SDS-PAGE with a mini-gel apparatus, blotted to nitrocellulose membranes and probed with either AIDS sera, normal serum, or biotin-labeled mouse monoclonal anti-FLAG M2 antibody, as indicated. M, MultiMark multi-colored standard (Invitrogen). Molecular weights are in kilodaltons.

The HIV-1 antisense gene sequence from +14–368 consisting of 354 bp was introduced, with no modifications, into a recombinant vector encoding an epitope tag sequence, DYKDDDDK, or FLAG (diagrammed in Figure 5A). Expression of FLAG, situated at the carboxyterminal end of the recombinant HIV-1 antisense gene-FLAG fusion protein would indicate RNA and protein product(s) were synthesized. The vector, itself, contains no productive signal for translation initiation upstream of FLAG, such that translation must initiate off RNA containing the inserted HIV-1 antisense gene sequences (Figure 4). Two vector constructs were made: one with the HIV-1 antisense gene linked to the carboxyterminal FLAG sequences (HIV-AS-FLAG), and a negative control vector with the same HIV-1 sequences in the opposite orientation, corresponding to nucleotides 87–441 of the LTR (REV) (Figure 5A). These two constructs, the empty vector as an additional negative control (-), or a control vector containing irrelevant DNA but the same pCMV-FLAG vector (C) used in construction of HIV-AS-FLAG or REV were separately transfected into HL2/3 cells. HL2/3 cells contain a molecular clone of HIV-1, HXB2/3 gpt, and express Gag, Env, Tat, Rev, and Nef, but not reverse transcriptase [31]. This cell line used in these experiments did not synthesize antisense RNA by RT-PCR and the HL2/3 proviral LTR was truncated by PCR analysis (data not shown). Because of the requirement for the HIV-1 Tat and Rev proteins for efficient transcriptional elongation and nuclear export for HIV-1 mRNAs, it was presumed these were also necessary to enable production of the HIV-1 antisense proteins. Following simultaneous control and HIV-AS-FLAG transfections, the cells were washed, lysed, and the lysate proteins were immunoaffinity-purified using mouse monoclonal anti-FLAG Ab (M2) affinity columns or resin split between lysate samples. The transfected lysate eluates were then concentrated.

Figure 5 demonstrates HAPs production. Given the finite HIV-1 DNA sequence of 354 bp of the HIV antisense gene inserted inbetween the CMV promoter of the vector and the multiple termination signals following the carboxyterminal FLAG sequences, the larger protein species selected on anti-FLAG Ab affinity resins can only be explained by multimerization. The choice of affinity tag, such as Myc, FLAG, or histidine, can strongly influence the oligomerization state of the resulting recombinant protein [32]. Oligomerization of HAPs was confirmed by Western blotting of the concentrated HIV-AS-FLAG eluates (Figure 5D, labeled anti-FLAG). Immunodetection of FLAG epitopes specifically identified proteins migrating as 53–54, 31–32, 27–28, and 24–25 kDa molecular mass bands, as well as fainter species ≈ 49, 44, and < 20 kDa. These were consistent with two of the predicted HAPs that contained the carboxyterminal FLAG epitope (diagrammed in Figure 3a and 3b). The expected molecular mass of the recombinant HIV-1 antisense protein including the FLAG epitope (AUG start) is ≈ 13.3 kDa; a dimer, trimer and tetramer would have a expected molecular mass ≈ 26,6, 39.9 and 53.2 kDa, respectively. The recombinant protein utilizing a CUG start would yield multimers of 13.7 kDa at ≈27.4, 41.1, and 54.8 kDa. The dimer and tetramer-sized molecular masses (migrating at 27–28 and 53–54 kDa) were especially prominent bands on multiple Westerns of HIV-AS-FLAG affinity-purified proteins probed with the directly-labelled mouse monoclonal antibody to the FLAG epitope (DYKDDDDK) (Figure 5D, anti-FLAG). To further add to the complexity, hetero-oligomerization could occur between the two larger HAP proteins (Figure 3a and 3b), or alternatively, between one of the larger HAP proteins (3a or 3b) and the shortest protein diagrammed in Figure 3c. The 81 amino acid protein predicted in the WT sequence of molecular mass 8.5 kDa would be slightly altered at the carboxyterminal end because of the recombinant vector sequences and is not in frame with the FLAG sequences. However, hetero-oligomerization between a and c (Figure 3) would yield dimer and tetramer forms of predicted 22 and 44 kDa molecular mass, respectively, which were bands repeatedly observed along with the 27–28 and 53–54 kDa species, both on silver-stained gels of HIV-AS-FLAG immunoaffinity-purified samples and, more faintly, on the anti-FLAG immunodetection (Figure 5D, anti-FLAG).

Of more particular significance is the prominent immunodetection of these same 44 and 54 kDa molecular mass proteins isolated from HIV-AS-FLAG immunoaffinity-purified samples on Western analysis by immunoglobulin purified from the sera of AIDS patients but not normal sera (Figure 5D, labelled AIDS sera and NORMAL sera).

Analysis of the immunoaffinity-purified HIV-AS-FLAG transfected cell lysates and control lysates was performed on a variety of gel types. These included Tris-glycine SDS-PAGE and Tris-Tricine-SDS-PAGE with the inclusion of 8 M urea in an attempt to adequately resolve the molecular masses of the proteins/polypeptides under 60 kDa in size (Figure 5B, mini-gel apparatus and 5C, large gel apparatus) [33,34]. Even following reduction with dithiothreitol (DTT) and alkylation with iodacetamide (Figure 5C, 5D, labelled D/I), the proteins remained highly oligomerized, possibly secondary to the FLAG tag, although the smaller predicted 13–14 kDa protein band was now observed (faintly) (Figure 5B, HIV-AS-FLAG). Control lysates from HL2/3 cells undergoing mock transfection (-), transfection with an irrelevant DNA/pCMV vector control (C) (Figure 5D), or transfection with the REV vector control (Figure 5C) did not produce any protein bands following immunoaffinity isolation, even with concentration in PEG (Figure 4B, 4D), providing the M2 affinity gel was performing adequately. This indicated that the HL2/3 cell line used in the transfection experiments detailed herein was not intrinsically capable of producing the HIV-AS-FLAG fusion protein(s) isolated on the M2 anti-FLAG antibody resins.

**Figure 6:**
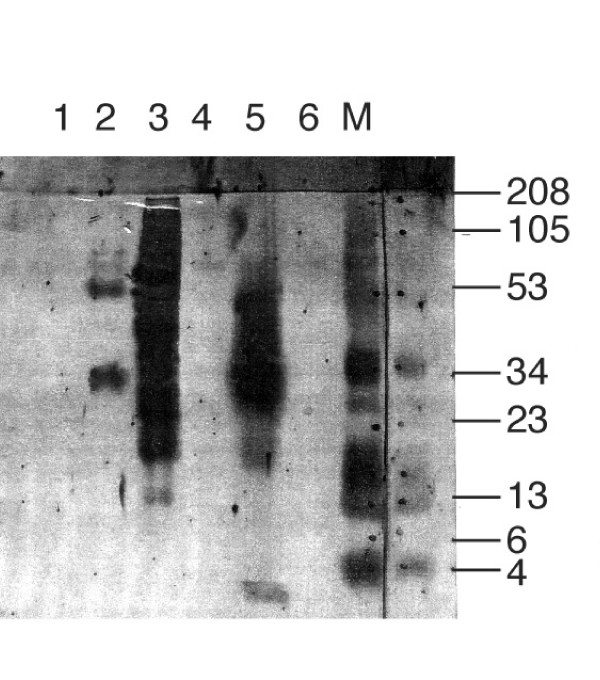
Antibody to an internal peptide (4041) of HIV antisense proteins detects proteins from HIV-AS-FLAG transfected cells, but not control-transfected cells on western blot analysis. Polyclonal rabbit antisera from rabbits immunized to peptide 4041 was affinity-purified over a 4041 peptide column (Biosource) and then used in immunoblot analysis. Peptide 4041 (+ control, lane 5), peptide 4040 (- control, lane 6), HIV-AS-FLAG transfected HL2/3 cell lysate (lane 3), and FLAG-affinity isolated eluates from HIV-AS-FLAG transfected (lane 2) vs empty vector transfected HL 2/3 cells (lane 1) were separated on Tris-tricine-urea SDS-PAGE, transferred to nitrocellulose membranes and the blots probed with anti-4041 antibody. M, MultiMark multi-colored standard. Molecular weights are in kilodaltons.

**Figure 7:**
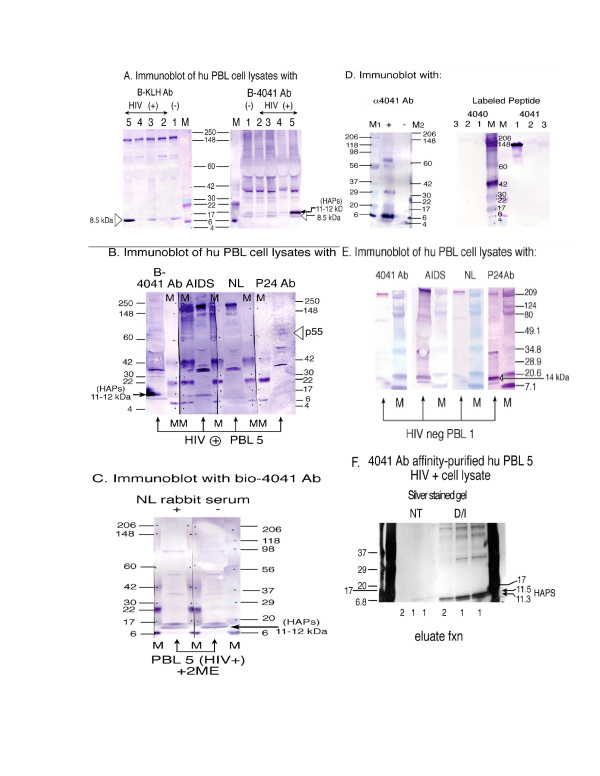
Detection and isolation of HAPs in human HIV+ peripheral blood lymphocytes (PBL) with antibody to an internal HAP peptide (4041). **(A) **Patients HIV (+) PBL 2–5 or HIV (-) PBL-1 cell lysates were fractionated by 4–20% tris-glycine SDS-PAGE and analyzed by Western blot with biotin-labeled rabbit anti-KLH antibody (B-KLH Ab) and with biotin-labeled rabbit anti-4041 antibody (B-4041 Ab). M, MultiMark multi-colored standard. **(B) **Cell lysates from HIV (+) PBL 5 were analyzed in replicate on 4–20% tris-glycine SDS-PAGE followed by Western blot using biotin-labelled rabbit anti-4041 Ab, or rabbit anti-p24 Ab, HIV Ig (labeled AIDS) and normal human sera, followed by biotin-labelled anti-rabbit Ig or anti-human Ig. **(C) **HIV (+) PBL 5 lysate was fractionated in the presence of 2 ME (as reducing agent) by 4–20% tris-glycine SDS-PAGE, transferred to membrane and probed with biotin-labeled anti-4041 Ab in the presence (+) or absence (-) of normal rabbit serum. M, MultiMark multi-colored standard. (**D**) The specificity of the anti-4041 Ab is demonstrated on immunodetection of the + peptide (4041), but not an unrelated peptide (4040, -). In the left panel, the anti-4041 Ab is used in immunoblotting detection of the peptides run in the gel and transferred to membrane. In the right panel, the anti-4041 Ab (lane 1) or unrelated antibodies (lanes 2,3) are separated on SDS-PAGE, transferred to membrane, and then probed with the biotin-labeled peptides, as indicated. (**E**) Cell lysates from HIV (-) PBL 1 were analyzed in replicate on SDS-PAGE followed by Western blot using rabbit anti-4041 Ab, rabbit anti-p24 Ab, HIV Ig (labeled AIDS) and normal human sera, as in (B). (**F**) Affinity-isolation of human (HIV +) PBL 5 cell lysates with HAP peptide 4041-specific antibody linked matrix. The affinity-purified anti-4041 Ab was cross-linked to coupling gel with 5 M sodium cyanoborohydride, as instructed (Pierce). Patient PBL 5 cell lysate (60 microliters) was incubated, in duplicate samples, with the anti-HAP peptide (4041) Ab linked gel in a spin column for 2 hours at RT, then the columns washed four times, and bound proteins eluted, as recommended (Pierce, Profound Mammalian Co-Immunoprecipitation Kit). Eluate fractions 1 and 2 from cell lysate samples that had been untreated (NT) or reduced and alkylated (D/I) were run in replicate on the same 4–20% tris-glycine SDS-PAGE. The gel was split along a marker lane (Biorad) and then silver stained (Invitrogen silver staining kit). The mass marker lanes on either end of the gel are MultiMark multi-colored standards (Invitrogen). Molecular weights are in kilodaltons.

To further identify HIV-1 antisense proteins (HAPs) in HIV-1 infected human cells, which would not have an epitope tag, it was imperative to develop antisera that would recognize HAP(s) internal peptide sequences. We had peptides synthesized, based on the derived amino acid sequences for HAPs, for immunization of rabbits, to enable testing of whether HIV antisense proteins were intrinsically produced by wild-type virus in human cells. Peptides were synthesized from immunogenic regions of each of the proposed translation products indicated in Figure 3 (arrowheads). One peptide, designated 4041 (amino acid sequence underlined in Figure 3 and boxed in Figure 4) was particularly successful in inducing antibodies in rabbits. Polyclonal rabbit antisera from two rabbits immunized with the 4041 peptide was affinity-purified over a 4041 peptide-linked column (Biosource). The affinity-isolated anti-4041 Ab reacted specifically with the 4041 peptide, but not a control peptide on dot blot and Western blot analysis (compare lane 5 (4041 peptide (+)) and lane 6 (4040 peptide (-)) Figure 6, Figure 7D). In addition, on Western blot analysis the anti-4041 Ab recognized protein bands migrating as 28–30 and 53–54 kDa molecular mass species from HIV-AS-FLAG transfected cells that had been affinity-purified on the M2 anti-FLAG Ab column and PEG concentrated but not the pCMVTag4A empty vector control-transfected HL2/3 proteins that had also been affinity-purified and PEG concentrated (compare lanes 1 and 2, Figure 6). Thus the affinity-purified proteins from the recombinant HIV antisense gene transfected cells, but not control transfected cells were recognized three ways by immunodetection on Western blots: the 53–54 kDa species was recognized by AIDS sera but not normal sera, by biotin-labelled mouse monoclonal antibody to the (entire) FLAG epitope, and by affinity-purified rabbit antibody to an internal HAP epitope, 4041. The latter two antibodies also recognized a 27–28 kDa species in affinity-purified HIV-AS-FLAG transfected proteins on these same blots.

### Identification and isolation of HAPs in human HIV+ peripheral blood lymphocytes (PBLs)

The directly biotin-labelled, affinity purified antibody to the internal HIV-1 antisense protein peptide 4041 was then used to probe human PBL cell lysates following 4–20% tris-glycine SDS-PAGE gel electrophoresis and transfer to membranes (Figure 7A, labelled B-4041 Ab). As a control, biotin-labelled rabbit anti-keyhole limpet hemocyanin (KLH) antibody was used in tandem to probe the same cell lysates (Figure 7A, labelled B-KLH Ab). This was a necessary control because the human lymphocyte cell lysates undoubtedly contained immunoglobulins (Ig) and other Ig-binding proteins. These would be capable of interacting with the immunoblotting antibody detection system following the renaturation that occurs upon transfer to a blot and washing. This capacity of antibody to recognize its antigen even following transfer to a blot from a denaturing gel is demonstrated with Figure 7D, where the rabbit anti-4041 antibodies (lane 1), but not control antibodies (lanes 2,3) bind specifically to the biotin-labelled 4041 peptide, but not a control biotin-labelled 4040 peptide (Figure 7D, compare lane 1 probed with 4041 peptide as opposed to 4040 peptide). Four HIV+ individual's PBL cell lysates, as well as a HIV negative PBL cell lysate control were analyzed by gel electrophoresis and then immunoblotted with either rabbit anti-KLH or rabbit anti-4041 antibody (Figure 7A). A protein band migrating at 8.5 kDa, appeared to react with both biotin-labelled antibodies and the interaction was judged to be non-specific (open arrowhead, Figure 7A). However, only immunodetection with the biotin-labelled anti-4041 Ab revealed two protein bands migrating at a molecular mass of 11.3 and 11.5 kDa from HIV positive PBL lysates, which were at the predicted molecular mass for the two larger HIV-1 antisense proteins of 105 and 108 aa in the wild-type virus (Figure 7A, 7C, labelled HAPs). Further analysis of this individual's HIV+ cell lysate proteins, run in replicate on the same gel and then simultaneously immunoblotted, showed that the 11.3/11.5 kDa species were recognized with the biotin-labelled rabbit anti-4041 antibody or AIDs antisera, but not normal sera, nor by control rabbit p24 antiserum (Figure 7B). Control HIV-negative human cell lysates (PBL 1) were also probed with the same antibody preparations and demonstrated no reactive bands at 11.3 or 11.5 kDa molecular mass (Figure 7E). However, antibody detection using both a primary and secondary labelled antibody universally had faint bands at 8–9 kDa and 14 kDa (Figure 7E).

Reducing disulfide bonds in the HIV+ cell lysate sample significantly removed the contribution of "non-specific" bands and allowed prominent detection of the HAPs 11.3 and 11.5 kDa molecular mass species with the affinity-purified antibody to the internal HIV antisense protein epitope (Figure 7C, immunoblot with biotin-labelled 4041 Ab). As an additional control, we used the affinity-purified antibody to the internal HAP epitope 4041 to perform affinity-isolation of the same human HIV+ PBL cell lysate. Once again the expected molecular mass bands of 11.3 and 11.5 kDa were prominent on the silver-stained gel, particularly when the eluate fractions were reduced and alkylated (D/I, Figure 7F).

## Discussion

This is the first demonstration of an antisense gene within the human immunodeficiency virus type 1 (HIV-1) LTR that functions to produce antisense protein(s) (HAPs) and an antisense transcript that originates from an initiator element in TAR region DNA, the HIVaINR (previously described in U.S. patents 5,919,677 and 6,392,029). The antisense transcript is intrinsically produced from the human immunodeficiency virus LTR template, shown both with *in vitro *transcription reactions and *in vivo*, in cultured human cell lines that have had the HIV-1 LTR sequences stably incorporated into the cellular chromosome (Figures 1C, 2B and additional File 2). Evidence is provided that the antisense RNA has the potential to be translated into protein(s) within human cells, including human PBLs (Figures 3, 4, 5, 6, 7). The HIV-1 antisense gene sequence corresponding to nucleotides 14–368 was incorporated into a eukaryotic expression vector that allowed addition of a carboxy terminal FLAG sequence tag. The FLAG-tagged HIV-1 fusion proteins (HAP-FLAG(s)) were then immunoaffinity purified on anti-FLAG Ab resins or columns. Western blots confirmed that the recombinant proteins, HAP-FLAG(s), form oligomers that are recognized by AIDS sera, anti-FLAG antibody, and antibody to an internal HAP peptide (anti-4041 Ab). Finally, this same anti-4041 Ab specifically recognizes the HAP wild-type species within HIV+ PBL cell lysates, but not HIV-control cell lysates, and could isolate HAPs. Each of these will be discussed in turn.

There are many remarkable aspects to this HIV-1 antisense RNA. One of us (LBL) previously suggested that, because of the potential for the beginning 25 nucleotides of the antisense RNA initiated from the HIVaINR to overlap, and form a complementary base-paired RNA duplex with the 5' end of HIV-1 sense transcripts, it could contribute to an intrinsic RNA regulatory system for HIV transcription and translation (see additional File 3 derived from U.S. patent 5,919,677). We suggested that the RNA duplex formation could modulate the effective synthesis of HIV sense transcripts by one or more mechanisms that might include inhibiting or attenuating efficient RNA polymerase II elongation of sense transcripts, altering the sense mRNA stability or processing in the nucleus, or inhibiting cap site function in initiation of translation (additional File 3 a-d). We also previously showed that the HIVaINR antisense RNA, when added to an HIV-1 LTR template linked to a bacteriophage promoter, could effectively abrogate detection of sense HIV RNA transcripts *in vitro *even with T7 RNA polymerase driving the sense transcription (U.S. patent 5,919,677, example 1, Figure 3). At the time, the most convincing models for naturally occurring antisense genes directing synthesis of antisense RNA capable of directly controlling gene expression were found in prokaryotes [35]. More recently, the discovery of RNA silencing by small interfering RNAs (siRNA), microRNAs (miRNAs), and repeat-associated small interfering RNAs (rasiRNAs) indicates that these small RNAs, 21–30 nucleotides in length, can mediate effects in many species, and may represent a more universal approach to gene regulation [36-41]. While the origins of the original dsRNA can vary from hundreds of base-pairs in length (siRNAs) to a more unstructured transcript (primary-miRNA) containing hairpin structures approximately 70 nt in length; [39] both pathways are convergent following processing in the nucleus and export to the cytoplasm. Both the processing of the long dsRNA into siRNAs and the pre-miRNAs into mature miRNAs require the dsRNA-specific endonuclease, Dicer, acting in conjunction with a dsRNA-binding protein, the tar-RNA binding protein (TRBP) in humans [39,42,43]. Subsequently, one of the small RNA strands in the miRNA duplex is loaded onto Argonaute 2 (Ago2), with the small RNA directing the cleavage of hundreds of target molecules, if there is extensive complementary base pairing of the small RNA to the target mRNA [39]. When siRNAs/miRNAs base pair only partially with the mRNA target, the function alters to blocking translation of the mRNA into protein.

Here we would like to propose the hypothesis that the HIVaINR antisense RNA, which has extensive secondary structure by M-fold analysis (not shown) including multiple stem-loop structures, might represent an intrinsic viral primary miRNA, with the capability of forming miRNAs directed to at least two major HIV-1 targets suggested by the literature [44,45]. The first target, as we previously proposed, would be the TAR RNA hairpin, which is present in all HIV-1 mRNAs (as well as in HIV-1 genomic RNA). The second target is additionally suggested by Bennasser, Y., et al., in their computer-directed analysis and identification of five candidate pre-miRNAs in HIV-1, and is an extensive stem-loop structure found in the nef mRNA[45]. The HIVaINR antisense RNA extends from the HIVaINR (in R) through the U3 region overlapping the nef gene ((additional File 1). It is exactly complementary to the nef mRNA sequence shown in the predicted miRNA precursor structure, is therefore also intrinsically capable of forming similar secondary structures, but would have the complementary sequence to nef mRNA and an extensively base-pairing final miRNA product predicted. Therefore, we would suggest that this may represent an intrinsic mechanism used by HIV-1 to silence gene expression from the HIV promoter in the absence of Tat protein, as well as additionally and specifically from nef mRNA; Ago2 bound HIVaINR antisense RNA-derived guide miRNAs directing cleavage at very specific sites in TAR RNA and nef mRNA. The HIV-1 Tat protein, which binds to the sense TAR RNA hairpin loop, as well as to the HIVaINR antisense RNA (LBL, unpublished data), can presumably alter the bound RNA secondary structures, such that the cleavage target is no longer accessible [46]. Tat protein, therefore, could be proposed to function as an anti-RNA silencer, in addition to its many other capabilities. The function of additional cellular TAR RNA binding proteins such as TRBP in this context remains to be determined [47]. These aspects will be focused on in more adequate detail in a separate paper. In this paper, we simply wanted to show that the HIV-1 LTR intrinsically produces an antisense transcript that initiates from the region of the HIVaINR, and which also encodes proteins. Human microRNAs can be derived from capped, polyadenylated transcripts that also function as mRNAs, and we believe that the HIVaINR antisense transcript also has the dual capability to code for protein as well as function (once processed) as miRNA[48].

Antisense RNA transcripts initiating from the region of the HIVaINR were easily detectable using a sensitive biotin-labelled RT-PCR that we developed (Figures 2 and additional File 2). We know that the RNA is initiated from the region of the HIVaINR and not from spurious sites elsewhere in the adjoining HIV-1 or cellular sequences, because alternative primers used to detect DNA contamination that would anneal to DNA slightly beyond the antisense RNA initiation site revealed no RT-PCR product (additional File 2). These results were confirmed by primer extension and RNase digestion, using a non-radioactive adaptation of both techniques that we developed (Figure 1 and data not shown). This HIV antisense transcript initiating from HIVaINR (HIVaINR-antisense transcript) is in contrast to what has been described in HTLV-1, where multiple initiation sites in the HTLV-1 3'LTR have been observed[49]. HIV-1 sense transcripts are more variably detectable, except when the RNA is obtained from stably-transfected cells that have been stimulated with calcium ionophore and PMA (Figure 2B). A low level of HIV-1 sense transcripts is consistent with many other studies suggesting that the HIV-1 promoter is regulated by attenuation or inefficient elongation in the absence of Tat[50].

Negative-strand RNA transcripts have previously been described in HTLV-1 and the lentiviruses, HIV-1 and feline immunodeficiency virus (FIV) [8,51-54]. A negative strand promoter (NSP) had been previously localized within the HIV-1 3' LTR U3 region[8]. This core NSP extended from -78 to -172 (from the HIV cap site) and corresponded to a region overlapping the NF-k B sites (ibid). Negative-strand ORFs, such as ASO1 first described by Miller[9], and the FIV antisense ORF and HIV-1 ASP [53,54] were also described as overlapping (but of opposing polarity) to the envelope gene, which is located well outside of the LTR in both FIV and HIV-1. Evidence for this previously described HIV-1 antisense protein, the ASP, was obtained by recognition of sera from HIV+, but not HIV-individuals of the in vitro translated, 189 amino acid, 19 kDa protein product[53]. It is of interest that both the FIV and the ASP antisense RNAs contained sequence complementary to both envelope and the highly-structured Rev-responsive element (RRE) [53-55]. This contrasts, however, with the HIV-1 antisense gene described in this paper. The HIV antisense gene described in this paper is located in U3-R of the LTR, contains the HIVaINR in TAR region DNA, and coding sequences that extend from the region complementary to the TATA box region through the U3 region, in the reverse orientation to the coding sequences for the other HIV proteins (Figures 3, 4 and additional File 1). In addition, the HIVaINR antisense RNA is complementary to the initial 25 nucleotide sequence of TAR RNA, which includes the Tat protein binding site. Inasmuch as both the 5'LTR and the 3'LTR contain identical sequence, there is no known impediment for this gene to be active in both regions.

We chose to begin looking for the proteins translated off the HIVaINR antisense RNA within human cells or human cell lines, because human cells are the target of HIV-1 infection, and the requirements for translation in human cells, as opposed to a bacterial expression system, can be very different. Another unique aspect of the HIV-1 antisense gene was the presence of stop codons within each reading frame (Figures 3, 4). Isolation of the HIV-AS-FLAG recombinant protein(s) indicates translational decoding must be occurring through these presumptive stop codons. The recombinant HIV-AS-FLAG vector was transfected into HL2/3 cells, cell lysates were prepared, and protein was purified using anti-FLAG affinity chromatography (Figures 5, 6). Affinity-purified protein product indicates two steps occurred: (1) an intact HIV-AS-FLAG RNA was synthesized *in vivo*, in the transfected human cells, and (2) translation of this recombinant HIV-1 antisense RNA through the carboxyterminal FLAG sequences occurred. The nucleotide sequence of the HIV-1 antisense gene inserted into the recombinant vector was repeatedly confirmed by sequencing, and both HAPs a (CUG start), and b (AUG start) contained UGA codons (Figures 3 and 4). Western blotting confirmed production of protein(s) containing the internal HAP epitope, in recombinant proteins isolated using anti-FLAG Ab affinity resins (Figure 6). In addition, a <14 kDa molecular mass gel band was removed from a denaturing gel (with affinity isolated HIV-AS-FLAG recombinant proteins as in Figure 5C), subjected to trypsin digestion, and the peptides analyzed by matrix-assisted laser desorption ionization-mass spectrometry (MALDI-MS) (data not shown). This analysis suggested that the predicted HAPs had been produced, with one of the peptide mass "fingerprints" from the <14 kDa band confirming translation through a portion of the FLAG epitope (Figure 3a and 3b, (11 aa) XXXX). Both the internal HAP epitope 4041 and the carboxy-terminal FLAG epitope present in HAPs a and b of the recombinant proteins are downstream of the UGA codons. It is intriguing that UGA codons can potentially encode selenocysteines, given the appropriate downstream secondary RNA structure, called a selenocysteine insertion sequence (SECIS)[30]. Eukaryotic selenoprotein synthesis requires a functional SECIS element linked downstream of an open reading frame: the specific sequence and structural features of SECIS elements required for directing selenocysteine incoroporation have been characterized[30]. We note that exactly 125 nucleotides downstream of the initial UGA found in HAPs a (CUG start) is a potential hairpin structure beginning with the required 5'**GUGA **(SECIS core), with a 9 bp lower stem (with bulges) and an adenosine hairpin loop structure containing **AAA **with 3 bp in the upper stem (see sequence below diagrammed 4041 peptide in Figure 4). However, the HIV-1 antisense RNA lacks the 3' **GA **sequence found in the consensus SECIS element structures, but instead contains a 3'GGG. RNA hairpin secondary structure is also important in ribosomal frameshifting to create the full-length gag-pro-pol precursor protein[56]. Regardless of the mechanism employed to read-through or "decode", the experiments outlined in this manuscript indicate that a translational recoding process is occurring and results in protein product(s). Translational decoding occurs in retroviruses, as well as other organisms, as a mechanism to strictly regulate the amount of protein product produced[28,56].

Isolation of the HAPs protein products also presented unique challenges. Expression of the HIV-1 antisense gene products had profound consequences for the transfected human Hela cell line, HL2/3. The eukaryotic vector CMV promoter driving HIV-1 antisense gene expression presumably was more efficient than the intrinsic HIV antisense promoter and led to accelerated cell death and apoptosis. Therefore protein isolation had to occur within a narrow time frame before the cells underwent apoptosis. The phenomenon of enhanced syncytium formation and apoptosis induced by the intrinsic HIV-1 antisense gene transfection (over control transfections) is the subject of a separate paper (U.S. patent 6,392,029 and data not shown). The immunoaffinity-purified (and concentrated) HIV-AS-FLAG recombinant proteins tended to oligomerize such that larger species than the 13.7 kDa or 13.3 kDa predicted for HAP-FLAG a or HAP-FLAG b following the anti-FLAG Ab affinity column were observed. This may have represented a function of the affinity purification utilizing the FLAG epitope, as has been previously described [32]. In addition some proteins tend to multimerize with concentration [57]. However, the oligomeric form may represent the functional form of the protein, as is suggested by the recognition of the HAP-FLAG tetrameric molecular mass species by AIDS sera (but not normal sera) (Figure 5D). There are many proteins that require oligomerization for function: transcription factors, TNF receptor ligands, HIV-1 envelope with gp-41 trimer and associated gp-120 trimer, to name a few [58]. There are a number of motifs in the predicted amino acid sequence that could facilitate interaction with other proteins, including a proline rich region containing PXXPXR at the amino-terminus in HAP b, and a leucine-rich region shared by HAPs a and b (Figure 4). Antibody to an internal HAP peptide (4041) recognized the expected molecular mass species in both HAP-FLAG transfected HL2/3 cells when the total cell lysate was examined (Figure 6, lane 3, expected sizes 13.3, 13.7 kDa) and in HIV+ PBL cell lysate (Figure 7A,B,C, expected sizes 11.3, 11.5 kDa), which were not present in controls or uninfected PBL cell lysates. Finally, immunoaffinity isolation of HIV+PBL lysate proteins on a resin stably linked to an antibody to the internal HAP peptide confirmed the expected molecular mass products of 11.3 kDa (HAP b) and 11.5 kDa (HAP a) (Figure 7F). Again the appropriate size products were apparent when the eluate sample was reduced and alkylated (Figure 7F, lanes labeled D/I).

The location of this HIV-1 antisense gene suggests its import in AIDS pathogenesis (see additional File 1). A particular group of long-term survivors with HIV-1 infection were found to have an attenuated virus with deletions in the Nef gene and the LTR [59]. The postulated original viral deletion was believed to be in the region of the LTR, which inspection reveals to be overlapping the HIV-1 antisense gene (see additional File 1B, common SBBC deletions: bracket at 9271–9317). Other studies have led to conflicting results with respect to the importance of the Nef gene[60,61]. Because the Nef gene overlaps the LTR and also the region encoding the 73 carboxy-terminal amino acids of HAPs, close inspection of the actual gene deletions associated with either progression to AIDS or nonprogression clearly reveals a line of demarcation with respect to the HIV antisense gene and HAPs (see additional File 1C). In the Brambilla, et al study, they documented large deletions in Nef associated with AIDS progression (but outside the HIV antisense gene coding region)[60]. However, regions of deletion in Nef corresponding to long term survival invariably included HAPs sequence encoding the carboxy-terminal 10–20 amino acids (please see additional File 1 detailing the genomic regions deleted in long term survivors with respect to the HAPs coding regions). It may be of interest that this carboxy-terminal sequence of the HIV antisense proteins includes 9/22 amino acids sharing identity with paromyxoviral fusion proteins, as follows[62].

HAPs(a/b) **P**LLP**YL**AQ**L**V**L**ACSTIQ**R**S**VDI**

Measles 437 **P**DAV**YL**HRID**L**GPPISLERL**D**V 459 F1

SV5 VTYNSTIK**L**ESSQILSIDPL**DI**

Sendai DATWGVQN**L**TVGPAIAI**R**P**VDI**

This would suggest that it is the previously unrecognized HIV antisense gene that is vital therefore for AIDS progression.

## Conclusion

A novel antisense gene within the HIV-1 LTR is described that intrinsically produces RNA and proteins (HAPs) and that may be of significance in AIDS pathogenesis. We would suggest that this gene may be of interest as a new target for vaccine development or AIDS intervention.

## Methods

### Human subjects and isolation of human peripheral blood lymphocytes (PBLs)

HIV + and HIV – blood donors were recruited from an Infectious Disease Clinic at Buffalo General Hospital, after informed consent, as approved by the institutional review board (State University of New York at Buffalo). Blood was diluted in Hank's balanced salt solution (HBSS) (Gibco BRL), layered on top of Histopaque 1077 (Sigma), and then centrifuged at 425 × g for 30 minutes at room termperature. The peripheral blood mononuclear cell (PBMC) layer was removed, washed three times with HBSS, and then resuspended in serum-free X-vivo 15 media (BioWhittaker) for incubation in a plate at 37°C in 5% CO_2_. After two hours of incubation, non-adherent cells were removed, washed in PBS, and then kept frozen in liquid nitrogen until experimental use, as described below. These cells were predominantly peripheral blood lymphocytes (PBLs).

### Cell lines, plasmids, and AIDS-specific reagents

The following reagents were obtained through the AIDS Research and Reference Reagent Program, Division of AIDS, NIAID, NIH: pHIV-CAT plasmid containing HIV-1 LTR U3 and R sequences 5' to the chloramphenicol acetyl transferase (CAT) gene from Drs. Gary Nabel and Neil Perkins[23]; the U38 and BF24 cell lines containing stably integrated and silent copies of the HIV-1 LTR-CAT sequences; the HL2/3 cell line containing stably integrated copies of the HIV-1 molecular clone HXB2/3gpt from Drs. Barbara K. Felber and George N. Pavlakis[24,31]; human HIV immunoglobulin (HIV-Ig), and rabbit p24 antiserum.

### In vitro transcription and RNA analysis

*In vitro *transcription reactions were performed with the indicated HIV-1 LTR DNA templates (Figure 1B) and *Drosophila *nuclear extracts as a source of eukaryotic RNA polymerases, as described by the manufacturer (*Drosophila *Embryo Nuclear Extract Transcription System II, Promega). The pooled and gel-purified DNA templates were generated by PCR using primers containing specific HIV-1 sequences (Figure 2A), as previously described [63]. Each *in vitro *transcription reaction was performed with 5 u *Drosophila *nuclear extract and the indicated DNA template, in 7.5 mM HEPES, pH 7.6, 60 mM K glutamate, 3.75 mM MgCl_2_, 1.5 mM DTT, 3% glycerol, 0.5 mM each rNTP, 2.5 μg/ml creatine kinase, 0.2 mM creatine phosphate, 6.25 μg/ml polyI poly C, and 10 u RNase inhibitor. Following incubation for 1 hr at 22°C, the reaction mixtures were DNase I treated, phenol-chloroform extracted and ethanol precipitated to purify the RNA synthesized *in vitro*. Primer extension reactions using biotinylated HIV-1 specific primers (Figure 1B) and 3 u AMV reverse transcriptase (RT) in a reaction buffer consisting of 50 mM Tris pH 8.3, 50 mM KCl, 10 mM MgCl_2_, 10 mM DTT, 1 mM each dNTP, 0.5 mM spermidine, and 2.2 mM Na-pyrophosphate were incubated for 60 min at 42°C[64]. A nuclear extract control, in which *in vitro *transcription was performed without added DNA template (no template; NT), was also analyzed by primer extension with either a sense primer (5'U3, Figure 1C, lane 4) or antisense primer specific for HIV sequences (3'510–530, Figure 1C, lane 11), and verified the lack of contaminating template contributed by the *Drosophila *nuclear extract or reaction components. Biotinylated primers utlized in primer extension reactions had the following sequences and are diagrammed in Figure 1B: 5' Ava I: TAATACGACTCACTATAGGGTTTCGTCACATGG-CCCGAGAGC; 5'AvaII:TAATACGACTCACTATAGGGGCATGGGATGGAGGACCCGGAG; 5'U3: TGGAAGGGCTAATTTGGTCC; and 3'510–530; TAATACGACTCACTATAGGGTTATTGAGGCTTAAGCAGTGG. (Additional, underlined sequence represents T7 site). Complementary DNA products were analyzed following electrophoresis on a 6% TBE-urea (denaturing PAGE) gel, transfer to Biodyne B membranes (Pall), and colorimetric detection, as previously described[63].

### Nonradioactive modification of RT-PCR and analysis of RNA synthesized in vivo

Human cell lines were expanded in culture with or without stimulation for 2 hours with calcium ionophore (A23187), 50 ng/ml, and phorbol myristate ester (PMA), 50 ng/ml. Total RNA was extracted from cells using TRIzol (Gibco BRL Life Technologies), and equally divided for separate treatments consisting of either two DNase I treatments (75 u, Gibco), or two DNase I treatments plus digestion with RNase A (70 μg/ml) and RNase TI (20 u), as described (Boehringer Mannheim Biochemicals: RNase Protection Kit). A proteinase K treatment (0.4 mg/ml) was essential following the initial DNase treatment. Nucleic acids were then purified by phenol-chloroform extraction and ethanol precipitation. **For antisense HIV-1 RNA detection**: the Ava1 primer complementary to antisense RNA was utilized (5'TTTCGTCACATGGCCCGAGAGC 3') during the reverse transcription step, then the Ava1 primer and a biotinylated 441 primer (5'CCAGAGAGACCCAGTACAGGCAAAA3') were utilized during PCR. These primers are illustrated in the context of the HIV antisense gene in Figure 4. **For sense HIV-1 RNA detection**: an antisense 3'CAT primer (5'CAAGAATGTGAATAAAGGC 3') (or alternatively 3'510–530 above) were used during RT, and 3'CAT (or 3'510–530) and biotinylated 5'472 primer (5'CAGATCTGAGCCTGGGA 3') were used during PCR. Reverse transcription was performed with 200 u MuLV-RT and the indicated primer in a reaction mixture containing 10 mM Tris, pH 8.3, 50 mM KCL, 2.5 mM MgCl_2_, 1 mM each dNTP, 40 u. RNase inhibitor, and 3 μl of the treated RNA. The reaction mixture was then incubated for 35 min at 42°C. PCR was performed, after adding the appropriate biotinylated primer, 0.2 mM each dNTP, and 2.5 U Taq DNA polymerase, for 30 cycles of denaturing, reannealing and extension at 94°, 45 sec; 60°C, 45 sec; 72°C, 2 min., as described (Perkin Elmer, RNA PCR Core Kit). The RT-PCR products were analyzed by 3% agarose gel electrophoresis, followed by transfer to Biodyne B membrane and colorimetric detection, as previously described [63].

The internal standard (IS+) RNA was synthesized from a DNA template containing the primer DNA sites used in RT-PCR, and used in each RT-PCR to enable monitoring of primer annealing efficiency and semi-quantitative comparison of different reaction mixtures. The DNA template was constructed by a series of overlapping, PCR amplification steps beginning with a core HIV-1 LTR template extending from PvuII to HindIII restriction enzyme sites (Figure 2A). Pooled PCR products were gel-purified, and used as the template for the next PCR reaction, which added in succession: AvaI and CAT primer sites, then T7-5'G3PDH and Sp6-3'G3PDH primer sites (Figure 2A). Either Sp6 RNA polymerase or T7 RNA polymerase was used to synthesize in vitro the antisense IS+ RNA or sense IS+ RNA standard, which was purified and quantitated spectrophotometrically. The shorter IS+ RNA contained the same primer sites used in RT-PCR, but yielded shorter products: the HIV-1 antisense IS+ RNA RT-PCR product was 59 bp, whereas the sense IS+ RNA RT-PCR product was 80 bp. A titration of input IS+ RNA was included as a control in each RT-PCR and analyzed along with the set of cell-derived RNA samples, using the identical primers and reaction components.

### Cell transfection, lysis, and analysis of protein following immunoaffinity column purification

HL2/3 cells [31] were grown to near confluency in 100 mm plates (~8 × 10^6 ^cells) and transfected with either the recombinant HIV-1 antisense gene-FLAG vector (HIV-AS-FLAG); the opposite orientation-HIV-1 sequences within the same vector (REV-FLAG) or empty vector (-) as a negative control; or a luciferase-FLAG vector as an additional control using Transfectam, as described by the manufacturer (Promega). Following transfection and incubation for 2 hr, complete media consisting of DMEM/F12 (Gibco BRL), 10% fetal calf serum, penicillin-streptomycin, 1 × nonessential amino acids and anti-PPLO (Gibco BRL) was added, along with Ca ionophore (50 ng/ml) and phorbol myristate acetate (PMA, 50 ng/ml), and the transfected cells were incubated overnight at 37°C in 5%CO2. Cells were washed in Tris-buffered saline supplemented with 1 mM CaCl_2_, and lysed with 1 ml lysis buffer per dish. Lysis buffer contained 25 mM Tris pH 7.4, 150 mM NaCl, 1 mM CaCl_2_, 1% Triton X-100, 27 μg/mL aprotinin, 10 μg/mL leupeptin, and 100 mM PMSF). Following incubation on ice for 30 min, cells were scraped, transferred to microtubes and spun at 15,400 × g to pellet cell debris. The cleared lysates from each set of transfections were purified over individual mouse monoclonal anti-FLAG M2 affinity columns, and eluted with 0.1 M glycine (pH 3.5), per the manufacturer (Sigma). Pooled eluates were concentrated in dialysis tubing (MWCO = 500) by packing in PEG 8000, and were then analyzed on Tris-tricine-urea PAGE gels with or without treatment with dithiothreitol (DTT) or DTT and iodoacetamide (D/I) [33]. Gels were electroblotted onto nitrocellulose membranes using an Xcell II blot module (Novex). The blots were blocked in either 1% BSA (for anti-FLAG Ab detection), 3% BSA/0.05% Tween-20 in TBS (for biotin-labeled anti-peptide antibody detection), or 5% non-fat dried milk/1% BSA, then directly probed with either biotinylated anti-FLAG M2 antibody (Stratagene), biotin-labeled rabbit anti-peptide antibody (Biosource), or indirectly probed with HIV-Ig (antisera) or normal sera, followed by biotinylated goat anti-human immunoglobulin (Fab_2_) (Biosource). Normal mouse sera was added to the immunodetection steps with human sera. Colorimetric detection of the blots was then performed, as described [63-65].

### Construction of recombinant HIV antisense gene-FLAG and REV-FLAG vectors

HIV-1 antisense gene sequences derived from pHIV-CAT[23] were generated by PCR, gel-purified, and cloned into pTarget, as described by the manufacturer (Promega). The inserted sequences were digested with BamH1 (site contributed by pTarget) and EcoRV, gel purified, and directionally subcloned into the pCMV-Tag4A vector (Stratagene) that had also been digested with BamH1 and EcoRV and gel-purified, using standard methods [64]. The recombinant HIV antisense gene-FLAG vector contains the CMV immediate early promoter upstream of the HIV antisense gene sequence, but no translation start signal except that intrinsic to the HIV antisense gene. In addition, the HIV antisense gene is linked to 9 amino acid sequences contributed by the vector in frame with the carboxy-terminal FLAG (DYKDDDDK) sequences and termination signals. REV-FLAG contains the identical HIV antisense gene insert sequences, but in the reverse orientation, within the pCMV-Tag 4A vector. Recombinant vector sequences were confirmed by sequencing.

## Abbreviations

Key Words: HIVaINR (Human immunodeficiency virus antisense initiator), HIV antisense gene, antisense transcription, HAPs (HIV antisense protein(s)), translational decoding, recombinant HAP-FLAG vector, selenocysteine insertion sequence (SECIS)

## Competing interests

Linda B. Ludwig (LBL) retains interest in U.S. patent 5,919,677 and patent applications 10/135,545, 11/020,772 and 11/076,728 as well as the relevant European patent applications (98921073.7; 99932605.3; 03252743.4) and Canadian patent applications (2,289,907 & 2,320,383). The State University of New York at Buffalo retains the patent rights to U.S. patent #6,392,029 (inventors: LBL, KAK, JLA).

## Authors' contributions

LBL conceived of the studies, developed the non-radioactive adaptations of primer extension and RT-PCR, supervised and analyzed the data, and wrote the manuscript. JLA participated in the design of studies (protein analysis) and helped to draft the manuscript. KAK helped with the construction of the recombinant HIV-AS-FLAG and REV vectors, performed transfections, RT-PCR, and protein isolation and analysis. SS and SB isolated PBLs and contributed to protein analysis. C-B H recruited HIV+ and HIV-patients for the studies of intrinsic HAPs production in PBL, and obtained informed consent. SAS provided support for the early studies and assisted with protein analysis.

## Supplementary Material

Additional File 1 **Figure 1S **Diagram of the HIV-1 genomic regions deleted in long term survivors with respect to the HIV antisense gene HAPs coding regions. Deletions in the nef/LTR are shown as empty (Figure 1B Long term survivors) or as cross-hatched regions (Figure 1C: comparison of nef mutations in long term survivors and progressors[60].) Click here for file

Additional File 2 **Figure 2S **RT-PCR analysis of Jurkat T cells transiently transfected with a vector containing the HIV-1 LTR or controls. From U.S. patent 6,392,029, example 3. Click here for file

Additional File 3 **Figure 3S **Proposed model for intrinsic RNA regulatory system for HIV transcription and translation (from U.S. patent 5,919,677). Click here for file
