# Supplementary figures and images for: Human Immunodeficiency Virus-Type 1 LTR DNA contains an intrinsic gene producing antisense RNA and protein products
Source: Retrovirology. 2006 Nov 8;3:80. doi: 10.1186/1742-4690-3-80 (PMC1654176; doi:10.1186/1742-4690-3-80)

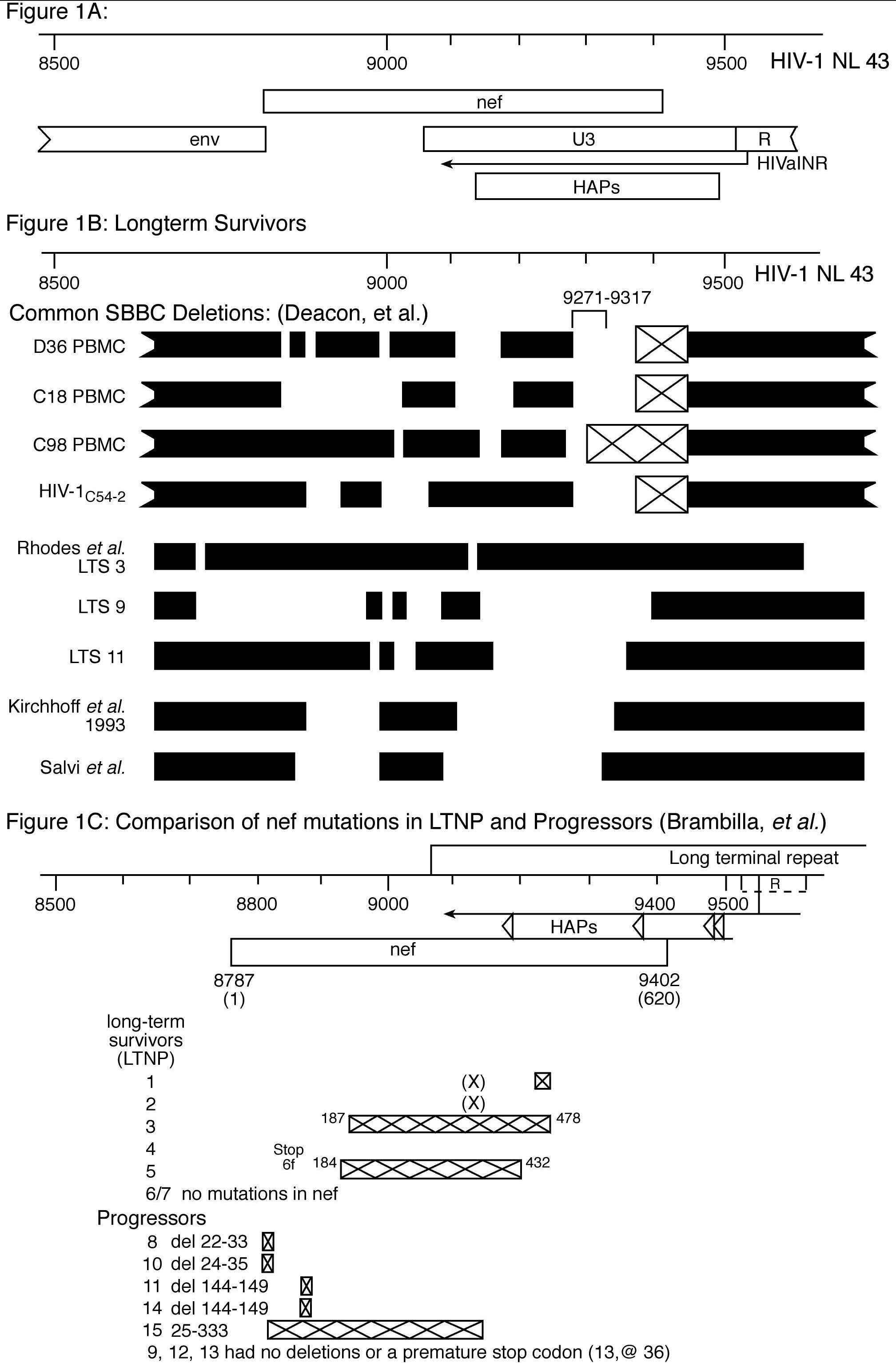

Supplement: Additional File 1 — Figure 1S Diagram of the HIV-1 genomic regions deleted in long term survivors with respect to the HIV antisense gene HAPs coding regions. Deletions in the nef/LTR are shown as empty (Figure 1B Long term survivors) or as cross-hatched regions (Figure 1C: comparison of nef mutations in long term survivors and progressors[60].) [file 1742-4690-3-80-S1.jpeg]

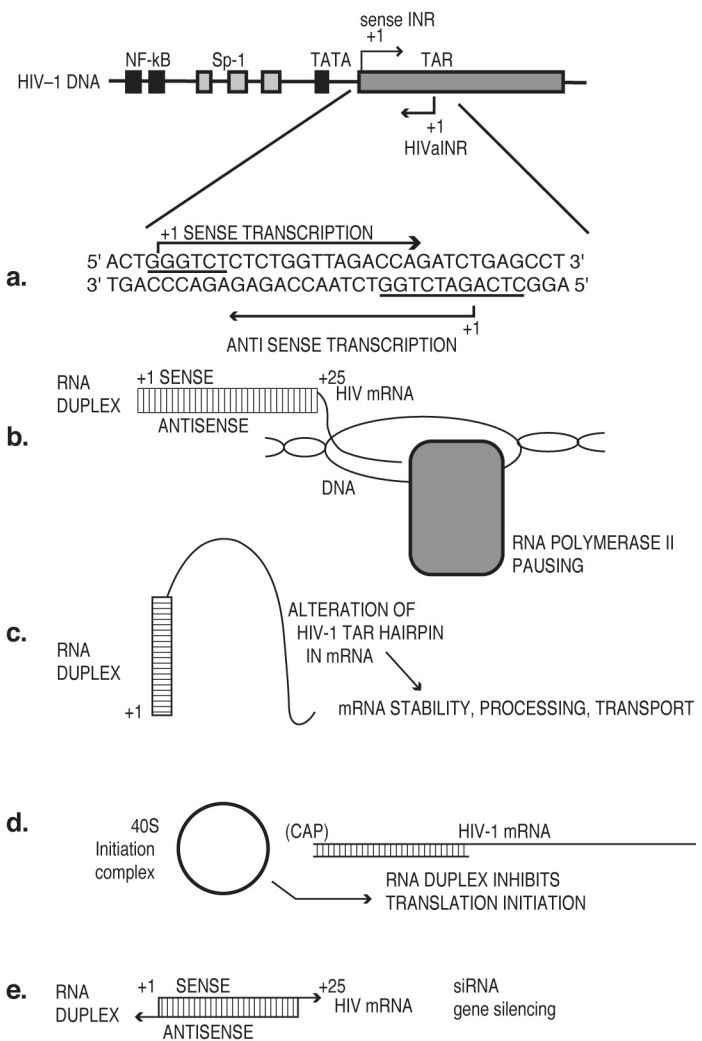

Supplement: Additional File 3 — Figure 3S Proposed model for intrinsic RNA regulatory system for HIV transcription and translation (from U.S. patent 5,919,677). [file 1742-4690-3-80-S3.pdf]
