# Supplementary material for: Human Immunodeficiency Virus-Type 1 LTR DNA contains an intrinsic gene producing antisense RNA and protein products
Source: Retrovirology. 2006 Nov 8;3:80. doi: 10.1186/1742-4690-3-80 (PMC1654176; doi:10.1186/1742-4690-3-80)
Supplement: Additional File 2 — Figure 2S RT-PCR analysis of Jurkat T cells transiently transfected with a vector containing the HIV-1 LTR or controls. From U.S. patent 6,392,029, example 3. [file 1742-4690-3-80-S2.pdf]

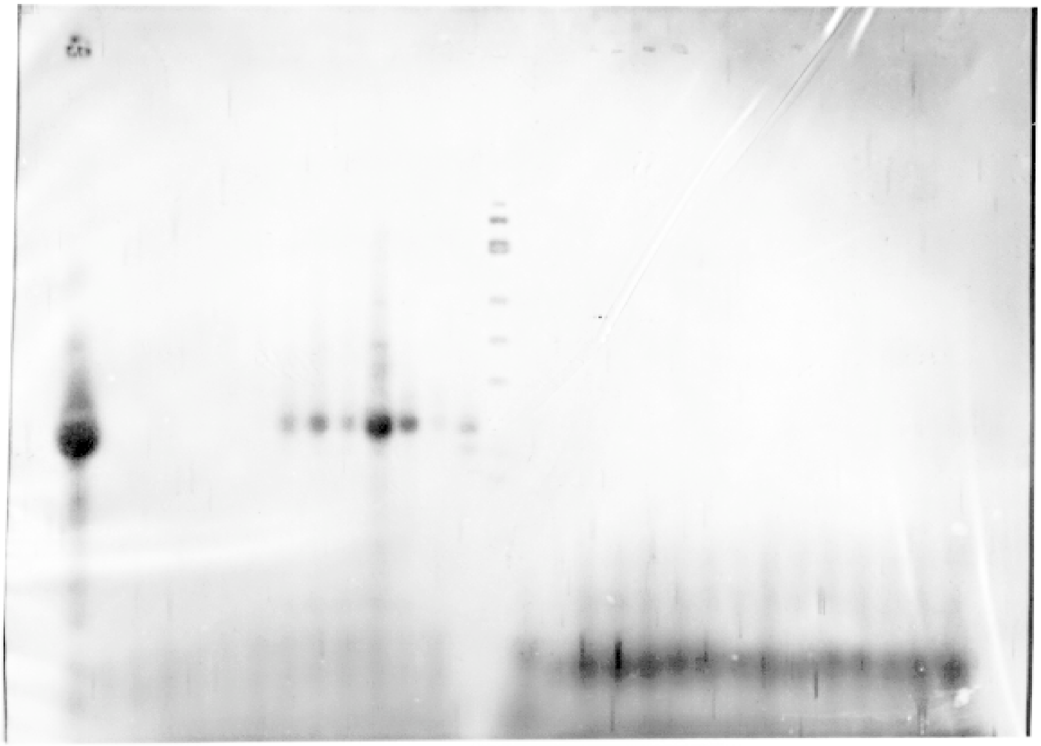
+ 1a 2a 3a 4a 5a 6a 7a 8a 9a 10a 11a 12a + M s- as- (-) 1b 2b 3b 4b 5b 6b 7b 8b 9b 10b 11b 12b
Jurkat T cells - + + + + + + + + + + + + + - M + + - + + + + + + + + + + + + +
RNA source Sp6 T7 Sp6
Transfection DNA - + + + - - - + + + + + + + + + + + - - - + + + + + +
NT
pSV-gal
pHIV-CAT
NT
pSV-gal
pHIV-CAT
DNase +
R.T. primer 5' Ava I 3'CAT 5' Ava I
PCR primers 5'Ava I / 3'441-b * ** 5' Ava I / 3'Mae I-b
Internal Standard + + s- as-(-)
